# When LHON Mimics Demyelination: Area Postrema Syndrome in Biallelic *DNAJC30* Variants

**DOI:** 10.3390/jcm15135289

**Published:** 2026-07-07

**Authors:** Kamil Dzwilewski, Magdalena Krygier, Jakub Szymarek, Marta Zawadzka, Urszula Stodolska-Koberda, Maria Mazurkiewicz-Bełdzińska

**Affiliations:** 1 Department of Developmental Neurology, Medical University of Gdansk, ul. Dębinki 7, 80-952 Gdansk, Poland; magdalena.krygier@gumed.edu.pl (M.K.); marta.zawadzka@gumed.edu.pl (M.Z.); maria.mazurkiewicz-beldzinska@gumed.edu.pl (M.M.-B.); 2 Department of Ophthalmology, Medical University of Gdansk, ul. Dębinki 7, 80-952 Gdansk, Poland; ukoberda@gumed.edu.pl

**Keywords:** *DNAJC30*, area postrema syndrome, LHONAR1, NMOSD

## Abstract

**Introduction**: Biallelic pathogenic variants in *DNAJC30* cause an autosomal recessive form of Leber hereditary optic neuropathy (LHONAR1), traditionally considered a mitochondrially transmitted disorder. The phenotypic spectrum of diseases linked to *DNAJC30* includes isolated optic neuropathy, Leigh syndrome spectrum (LSS), and atypical LHON-plus. **Case description**: Here, we report a 13-year-old boy presenting symptoms of area postrema syndrome (APS), with recurrent vomiting, vertigo, nystagmus, and subacute visual deterioration with central scotoma. Ophthalmological examination revealed bilateral papilledema with telangiectatic vessels, while visual evoked potentials demonstrated severe bilateral optic pathway dysfunction. Brain magnetic resonance imaging (MRI) showed T2/FLAIR hyperintense lesions involving the area postrema and enhancement of the optic nerves, strongly suggesting seronegative neuromyelitis optica spectrum disorder (NMOSD). Extensive immunological and cerebrospinal fluid studies, including anti-aquaporin-4 (AQP4) and anti-MOG antibodies, were negative. High-dose corticosteroids and intravenous immunoglobulins resulted in only transient and incomplete improvement, followed by further visual decline. Additionally, laboratory tests detected elevated lactate plasma levels. Hence, whole-exome sequencing was performed, which identified a homozygous pathogenic *DNAJC30* c.152A>G, p.(Tyr51Cys) variant, associated with LHONAR1. After initiation of idebenone therapy, the patient showed significant improvement in visual function, normalization of lactate levels, and complete resolution of the brainstem lesions on follow-up MRI. **Conclusions**: This case further expands the neuro-ophthalmic spectrum associated with *DNAJC30* variants and suggests that *DNAJC30*-related disease may closely mimic seronegative NMOSD. We highlight that early genetic diagnosis is essential, as recognition of this mitochondrial etiology enables targeted therapy and may substantially improve clinical outcomes.

## 1. Introduction

The auxiliary role of the DNAJC30 protein in mitochondrial function was first described by Tebbenkamp et al. in 2018, who demonstrated that it promotes energy production by interacting with ATP synthase subunits [1]. In 2021, Stenton et al. reported that biallelic pathogenic variants in the nuclear-encoded gene *DNAJC30* cause an autosomal recessive form of Leber hereditary optic neuropathy (LHONAR1), a condition previously considered to be exclusively mitochondrially inherited [2]. *DNAJC30* alterations lead to mitochondrial dysfunction via impaired complex I (CI) repair mechanisms. Notably, the Eastern European population exhibits a high prevalence of the *DNAJC30* c.152A>G, p.(Tyr51Cys) variant, likely due to the founder effect [2,3]. Further research expanded the phenotypic spectrum of diseases linked to *DNAJC30*. Although most affected individuals present with isolated optic neuropathy, several cases with features of Leigh syndrome spectrum (LSS) have been described [3,4,5,6,7]. In addition, recently, Şenol et al. reported a patient with an atypical LHON-plus, who was initially misdiagnosed with antibody-negative neuromyelitis optica spectrum disorder (NMOSD) with symptoms of area postrema syndrome (APS). However, following whole-exome sequencing (WES), which identified a homozygous *DNAJC30* c.352G>T, p.(Glu118Ter) variant, the diagnosis was revised to LHONAR1 [8]. These studies suggest that DNAJC30 mutations are linked to a spectrum of clinical outcomes.

Here, we report a second patient with LHON and APS, further supporting that *DNAJC30*-associated disease may mimic NMOSD. Additionally, we discuss the expanding neuro-ophthalmic manifestations associated with *DNAJC30* mutations.

## 2. Case Description

A 13-year-old patient of Polish origin, with normal psychomotor development and an unremarkable family history, was referred to the Department of Developmental Neurology due to recurrent vomiting and vertigo for 2 months prior to admission. Initially, symptoms were induced by physical activity, but later they intensified and occurred several times per day without any trigger, which led to 6 kg weight loss. Additionally, the patient experienced vision impairment (blurred vision with central scotoma) of the left eye. On admission, he presented horizontal gaze-evoked nystagmus and convergence insufficiency of the left eye without any other abnormalities on neurological examination. Ophthalmological evaluation showed bilateral papilledema with telangiectatic vessels and significant vision impairment of the left eye. Visual evoked potentials (VEPs) revealed a significant bilateral conduction disturbance in the visual pathway (left eye: latency Pmax 185 ms, amplitude 3.0 uV; right eye: latency Pmax 94.5 ms, amplitude 1.17 uV). Brain magnetic resonance imaging (MRI) identified T2/FLAIR high-signal lesions, without diffusion restriction in the brain stem, on the border of the pons and the medulla oblongata, of suspected inflammatory etiology (Figure 1). MRI of the spinal cord was normal. Laboratory tests did not detect antibodies against Lyme disease, antinuclear antibodies (ANA), cytoplasmic anti-neutrophil cytoplasmic antibodies (c-ANCA), perinuclear anti-neutrophil cytoplasmic antibodies (p-ANCA), anti-aquaporin-4, anti-myelin oligodendrocyte glycoprotein (anti-MOG), or autoimmune encephalitis. Cerebrospinal fluid (CSF) examinations did not display any abnormalities, and the CSF oligoclonal bands were negative. Suspecting an inflammatory etiology of the lesions, 5-day treatment with methylprednisolone was commenced at a daily dose of 1.0 g. Due to a lack of improvement, intravenous immunoglobulin (IVIG) therapy was initiated, resulting in a reduction in vomiting, vertigo, and slight improvement in vision. Follow-up brain MRI showed T2/dark fluid high-signal, non-contrast-enhancing lesions in the area postrema and enhancement of optic nerves, indicative of NMOSD (Figure 1). Oral steroid therapy was subsequently introduced. Unfortunately, despite immunosuppressive treatment, visual function did not improve. On the contrary, the patient started to present progressive vision impairment of the right eye with central scotoma. Additionally, laboratory tests detected an elevated lactate plasma level (up to 4.9 mmol/L).

WES was performed in an external laboratory, Blueprint Genetics (Espoo, Finland), on genomic DNA extracted from whole blood. WES consisted of sequence analysis of all protein-coding genes, coupled with copy number variation analysis, as well as mitochondrial genome analysis. The test targets all protein-coding exons, exon-intron boundaries (±20 bps), and selected noncoding, deep intronic variants. Sequencing performance metrics included a median coverage of 152×, with 99.77% of target bases covered at ≥20×. Mitochondrial sequencing achieved a median coverage of 3836×, with 100% of the mitochondrial genome covered at ≥1000×. Detailed information on sequencing, interpretation, and confirmation methods is available online at https://blueprintgenetics.com (accessed on 22 June 2026).

WES identified a homozygous *DNAJC30* variant c.152A>G, p.(Tyr51Cys) (NM_032317.3). No other pathogenic or likely pathogenic variants in established disease genes, including mtDNA genes, were identified in the patient. The c.152A>G variant is classified as pathogenic or likely pathogenic according to ACMG/AMP criteria [9]. The variant fulfills the following evidence criteria: PS3 (well-established in vitro and in vivo functional studies showing a deleterious effect on mitochondrial complex I activity), PM3 (identified in a homozygous state in multiple unrelated affected individuals with an autosomal recessive phenotype), PM2 (low frequency in population databases), and PP3 (multiple lines of computational evidence predicting a deleterious effect on the gene product).

The patient was diagnosed with LHONAR1 with symptoms of APS and provided with idebenone treatment. His recent ophthalmological examination after 10 months of treatment showed a significant vision improvement in both eyes (visual acuity right eye (VAR): 0.16, visual acuity left eye (VAL): 0.7) in comparison to the last evaluation before treatment (VAR: 0.08, VAL: 0.08). Laboratory tests revealed normalization of lactate plasma level. Control brain MRI revealed full regression of previously detected lesions (Figure 1). Figure 2 presents a chronological overview of the major clinical events, diagnostic procedures, therapeutic interventions, and follow-up findings in the patient’s disease course.

## 3. Discussion

Stenton et al. first described the autosomal recessive inheritance pattern of LHON, identifying 29 patients homozygous for the *DNAJC30* p.(Tyr51Cys) variant. All patients exhibited the characteristic LHON triad: circumpapillary telangiectatic microangiopathy, tortuosity of the central retinal vessels without fluorescein leakage, and pseudoedema of the retinal nerve fiber layer [2].

Similarly, Kieninger et al. identified *DNAJC30* mutations in 35 individuals with LHON or other optic atrophies. The p.(Tyr51Cys) allele accounted for 90% of cases, and two additional pathogenic variants were identified: the nonsense variant c.610G>T, p.(Glu204*), and the in-frame deletion c.230_232del, p.(His77del) [10]. It should be noted that LHONAR1 patients are indistinguishable from mitochondrially inherited LHON (mtLHON) on ophthalmological examination [3]. Furthermore, not all triad symptoms may be present in every patient, and in approximately 20% of patients, the ocular fundus may appear normal during the acute phase of LHON [10,11]. LHONAR1 is associated with earlier age of onset and a higher rate of visual recovery, both spontaneous and with idebenone, compared with mtLHON. Penetrance of biallelic *DNAJC30* variants is estimated at ~95% in males and ~60% in females, with marked male predominance (9:1) [3].

Importantly, most reported LHONAR1 cases to date originate from Poland, Russia, Ukraine, and Romania. In an in-house database of >4500 WES datasets from individuals of Polish ancestry, the frequency of the c.152A>G allele was estimated at 0.011 [4]. However, the exact prevalence of LHONAR1 remains unknown and is likely influenced by incomplete penetrance. In a study by Skorczyk-Werner et al., which analyzed a cohort of 46 Polish individuals with LHON who tested negative for common mtDNA mutations, all patients were found to carry biallelic *DNAJC30* variants. The p.(Tyr51Cys) variant was present in every case, either in a homozygous or compound heterozygous state. The authors compared the number of mtLHON diagnoses at a single genetic center (102 patients over 15 years) with the number of LHONAR1 diagnoses (32 patients over two years), concluding that LHONAR1 may be more prevalent than mtLHON in Poland. These results support prioritizing *DNAJC30* testing as a first-line diagnostic approach for patients with suspected LHON [11].

While LHONAR1 manifests mostly as isolated optic atrophy, several individuals with LSS were reported. LSS is a rare mitochondrial disease characterized by symmetrical lesions in the basal ganglia or brain stem with lactate peak on MRI, and a gradual deterioration of cognitive and motor functions. LSS is linked to variants in multiple mitochondrial DNA and nuclear genes, often affecting CI function [12]. To date, eight patients with biallelic *DNAJC30* variants have shown the LSS phenotype [3,4,5,6,7]. The mechanism driving LSS rather than isolated optic neuropathy remains unclear. A proposed digenic model suggests that LSS arises from an LHON-associated variant combined with a heterozygous damaging variant in another CI-encoding nuclear gene [13]. Supporting this, the coexisting rare heterozygous missense variants in *NDUFS8*, *NDUFS2*, or *NDUFA9* were found in six of the eight *DNAJC30*-associated LSS cases [3,4,5].

The phenotype spectrum from isolated LHON and LHON-plus to LSS is described for several complex I–related genes [14]. As for *DNAJC30*, there is an emerging literature on LHON-plus phenotypes, characterized by symptoms of acute optic neuropathy with concurrent acute brainstem lesion [8]. The area postrema (AP), located on the floor of the fourth ventricle, integrates chemical and neural inputs and serves as the brainstem’s “vomiting center.” APS arises from lesions in this AQP4-rich region and is often an early manifestation of NMOSD [15]. AQP4 antibody binding in the AP appears to downregulate, rather than activate, complement, disrupting water and neurotransmitter homeostasis and triggering nausea and vomiting. Importantly, a substantial proportion of patients with APS are seronegative for AQP4 antibodies. LHONAR1 may be considered in selected patients presenting with APS and NMOSD-like features who are seronegative for AQP4 antibodies, particularly when accompanied by poor response to steroid therapy and evidence of mitochondrial dysfunction. Elevated blood lactate levels and the presence of a lactate peak on MRI during the acute phase may further support consideration of LHONAR1 and prompt genetic testing for *DNAJC30* variants. Moreover, NMOSD patients do not present the characteristic LHON triad in ophthalmological examination, which may be useful in the differential diagnosis. Establishing a diagnosis is crucial, as it allows consideration of idebenone therapy. Although significant visual improvement was observed in our patient, this is a single case report and therefore does not provide sufficient evidence to attribute the outcome to idebenone with confidence. The observed improvement may also have been influenced by prior treatments and the possibility of spontaneous recovery.

Finally, the coexistence of multiple sclerosis (MS)and LHON, occurring more frequently than expected by chance, is referred to as Harding’s syndrome (LHON-MS), suggesting that LHON-associated mitochondrial variants may predispose individuals to MS or influence its progression [16]. However, it remains unclear to what extent the lesions primarily reflect mitochondrial dysfunction or inflammatory processes. We hypothesize that some cases classified as LHON-MS may in fact represent LHON-plus, with more extensive neuronal damage driven by mitochondrial dysfunction. Given the expanding phenotypic spectrum of *DNAJC30*-associated disease, it is possible that some LHON-MS cases arise from biallelic *DNAJC30* mutations, alone or in combination with other complex I–encoding nuclear or mitochondrial variants. Further studies are needed to explore this potential association.

## 4. Conclusions

In conclusion, *DNAJC30* variants may cause LHON with brainstem involvement mimicking seronegative NMOSD. Our case supports the need to consider *DNAJC30*-related disease in patients with optic neuropathy and area postrema syndrome who do not respond to immunotherapy. Early genetic diagnosis is essential, as targeted treatment with idebenone may lead to significant clinical and radiological improvement.

## Figures and Tables

**Figure 1:**
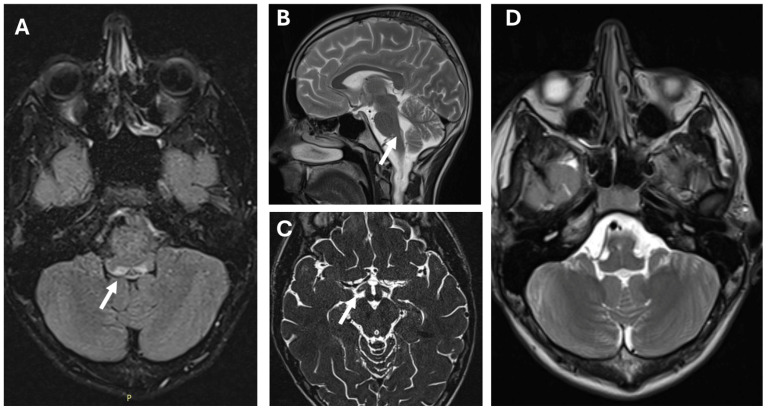
Brain MRI findings in a 13-year-old patient with symptoms of area postrema syndrome. (**A**) T2 dark fluid lesions without diffusion restriction in the brainstem, on the border of the pons and the medulla oblongata; (**B**) T2 high-signal lesions in the area postrema; (**C**) enhancement of the right optic tract in T2 sequence; (**D**) full regression of previously detected lesions in T2 sequence.

**Figure 2:**
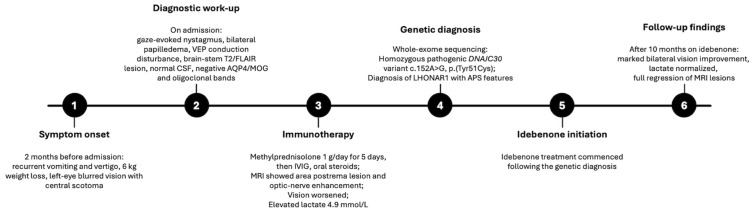
Timeline of the patient’s clinical course.

## Data Availability

The original contributions presented in this study are included in the article. Further inquiries can be directed to the corresponding authors.

## Abbreviations

The following abbreviations are used in this manuscript:

LHONAR1	Leber-like hereditary optic neuropathy, autosomal recessive 1
LSS	Leigh syndrome spectrum
APS	Area postrema syndrome
MRI	Magnetic resonance imaging
NMOSD	Neuromyelitis optica spectrum disorder
AQP4	Aquaporin-4
CI	Complex I
WES	Whole-exome sequencing
VEP	Visual evoked potentials
ANA	Antinuclear antibodies
c-ANCA	Cytoplasmic anti-neutrophil cytoplasmic antibodies
p-ANCA	Perinuclear anti-neutrophil cytoplasmic antibodies
Anti-MOG	Anti-myelin oligodendrocyte glycoprotein
CSF	Cerebrospinal fluid
IVIG	Intravenous immunoglobulin
VAR	Visual acuity right eye
VAL	Visual acuity left eye
mtLHON	Mitochondrially inherited LHON
MS	Multiple sclerosis
LHON-MS	Harding’s syndrome

## References

[1] Tebbenkamp A.T.N., Varela L., Choi J., Paredes M.I., Giani A.M., Song J.E., Sestan-Pesa M., Franjic D., Sousa A.M., Liu Z.-W. (2018). The 7q11.23 Protein DNAJC30 Interacts with ATP Synthase and Links Mitochondria to Brain Development. Cell.

[2] Stenton S.L., Sheremet N.L., Catarino C.B., Andreeva N.A., Assouline Z., Barboni P., Barel O., Berutti R., Bychkov I., Caporali L. (2021). Impaired complex I repair causes recessive Leber’s hereditary optic neuropathy. J. Clin. Investig..

[3] Stenton S.L., Tesarova M., Sheremet N.L., Catarino C.B., Carelli V., Ciara E., Curry K., Engvall M., Fleming L.R., Freisinger P. (2022). DNAJC30 defect: A frequent cause of recessive Leber hereditary optic neuropathy and Leigh syndrome. Brain.

[4] Zawadzka M., Krygier M., Pawłowicz M., Wilke M.V.M.B., Rutkowska K., Gueguen N., Desquiret-Dumas V., Klee E.W., Schimmenti L.A., Sławek J. (2022). Expanding the phenotype of DNAJC30-associated Leigh syndrome. Clin. Genet..

[5] Nesti C., Ticci C., Rubegni A., Doccini S., Scaturro G., Vetro A., Guerrini R., Santorelli F.M., Procopio E. (2023). Additive effect of DNAJC30 and NDUFA9 mutations causing Leigh syndrome. J. Neurol..

[6] Shen C., Wang K., Li W., Serrano A., Powers K., Zhang C., Chen J., Sun M. (2023). A homozygous nonsense mutation in DNAJC30 causes Leber’s hereditary optic neuropathy with Leigh-like phenotypes. Genes Dis..

[7] Dzwilewski K., Chojnowski K., Krygier M., Zawadzka M., Chylińska M., Mazurkiewicz-Bełdzińska M. (2024). Effects of idebenone treatment in a patient with DNAJC30-associated Leigh Syndrome. Neurol. Neurochir. Pol..

[8] Şenol H.B., Soydemir D., Polat A., Aydın A., Hız A.S., Yiş U. (2025). An Unusual Presentation of Leber Hereditary Optic Neuropathy-Plus Case Caused by a Novel DNAJC30 Variant. Am. J. Med. Genet A.

[9] Richards S., Aziz N., Bale S., Bick D., Das S., Gastier-Foster J., Grody W.W., Hegde M., Lyon E., Spector E. (2015). Standards and guidelines for the interpretation of sequence variants: A joint consensus recommendation of the American College of Medical Genetics and Genomics and the Association for Molecular Pathology. Genet Med..

[10] Kieninger S., Xiao T., Weisschuh N., Kohl S., Rüther K., Kroisel P.M., Brockmann T., Knappe S., Kellner U., Lagrèze W. (2022). *DNAJC30* disease-causing gene variants in a large Central European cohort of patients with suspected Leber’s hereditary optic neuropathy and optic atrophy. J. Med. Genet..

[11] Skorczyk-Werner A., Tońska K., Maciejczuk A., Nowomiejska K., Korwin M., Ołdak M., Wawrocka A., Krawczyński M.R. (2023). *DNAJC30* Gene Variants Are a Frequent Cause of a Rare Disease: Leber Hereditary Optic Neuropathy in Polish Patients. Int. J. Mol. Sci..

[12] Baertling F., Rodenburg R.J., Schaper J., Smeitink J.A., Koopman W.J.H., Mayatepek E., Morava E., Distelmaier F. (2014). A guide to diagnosis and treatment of Leigh syndrome. J. Neurol. Neurosurg. Psychiatry.

[13] Blickhäuser B., Stenton S.L., Neuhofer C.M., Floride E., Nesbitt V., Fratter C., Koch J., Kauffmann B., Catarino C., Schlieben L.D. (2024). Digenic Leigh syndrome on the background of the. Brain.

[14] Lenaers G., Beaulieu C., Charif M., Gerber S., Kaplan J., Rozet J.M. (2023). Autosomal recessive Leber hereditary optic neuropathy, a new neuro-ophthalmo-genetic paradigm. Brain.

[15] Chan K.H., Vorobeychik G. (2020). Area postrema syndrome: A neurological presentation of nausea, vomiting and hiccups. BMJ Case Rep..

[16] Rahimi G., Silverman M., Lucas M., Kazerooni L., Yousuf M.M., Jafarpour S., Santoro J.D. (2025). Leber’s hereditary optic neuropathy and multiple sclerosis: Overlap between mitochondrial disease and neuroinflammation. Front. Neurol..

